# How to Use a Repertory*The above article, from the pen of the former editor of this journal, is in answer to a letter of Dr. Yingling asking for an article upon the use of the Repertory.—Eds.

**Published:** 1891-01

**Authors:** 


### HOW TO USE A REPERTORY.*

Editors Homœopathic Physician:

In answering the queries of your correspondent, it must be remembered that each physician has his own way of doing his own work; hence answers to his queries can only be given in a general way. Upon these four points, he asks suggestions:

1. The Abuse of Repertories.

---

* The above article, from the pen of the former editor of this journal, is in answer to a letter of Dr. Yingling asking for an article upon the use of the Repertory.—Eds.

2. The Use of Repertories.
3. The Comparison of Repertories.
4. The Physician's Repertory.

The first two points may be considered together; for if a repertory be used rightly it cannot be abused. It must be remembered that a repertory is really only an index to the *Materia Medica*, not in any sense a separate work upon that subject. It is an index which tells one where to look in the *Materia Medica* for any given subject. The *Materia Medica* itself is very defective and hence all indices, or repertories, to it must necessarily be also defective. One cannot safely prescribe for his patients by simply looking up his symptoms in a repertory. He should find in the repertory the remedy, or the remedies, most nearly indicated and then look over the *Materia Medica* to be sure of his prescription. To prescribe by looking up a few symptoms in a repertory, without any reference to a *Materia Medica*, would be an abuse of the repertory, and the result apt to be a failure.

Before attempting to use a repertory correctly it is essential that the prescriber should know how to prescribe correctly. He should know how to examine a patient properly and record such symptoms; also how to select the few peculiar symptoms from the many common ones. His prescription must be based upon the symptoms which are peculiar to the individual case under consideration; these are the ones to be sought for in the repertory.

Each case has a few peculiar symptoms upon which the prescription must be made; the many general symptoms do not count in selecting the remedy. Suppose the patient complains of a cough; one would not think of looking in a repertory to see what remedies have a "cough," it is too common. But suppose further, the patient complains of being aroused from sleep at some special hour by his cough; on referring to a repertory, it may be that only a few remedies have a cough arousing one at that special hour. Further questioning may elicit the information that the cough is accompanied by some peculiar pain in head or in chest; on reference to the repertory, we may find that only one or two remedies are given as having this symptom and the previous one also. Now, our use of the repertory has narrowed down to, say two or three, the remedies we must study in the *Materia Medica* to ascertain the one, true simillimum. A further examination of the patient ought easily to elicit sufficient data to enable one to decide with some degree of certainty which remedy he should *study* in his *Materia Medica*, not which remedy he should prescribe off-hand.

The old pioneers in Homœopathy used to "thumb" their *Materia Medica*, running it over from Aconite to Zinc, to find the remedy having the peculiar symptoms of their patients. We, fortunately, having better repertories, do not need to do this in each case. Yet one cannot doubt but that this "thumbing" of the *Materia Medica* made our predecessors thorough students of materia medica and gave them that success which has given Homœopathy its proud title of being *the healing art*. Repertories are most certainly abused when one uses them to prescribe from instead of studying the *Materia Medica*.

The first symptom to be sought for in a repertory ought to be the most peculiar one; the one which is probably to be found under only a few drugs. The second symptom to be looked for should be the next "most uncommon;" and so on. By following this method, one generally needs to look for only three or four symptoms before he is ready to open his *Materia Medica*. As a general rule, we find that the mental symptoms are the most peculiar and so the best ones to be looked up first. Each remedy has its general characteristic symptoms and its peculiar local or pathological ones; each remedy has its peculiar headache or cough or stool and its general characteristics. So in prescribing, say for a case of headache, we must find a remedy having the local symptoms of the head—plus the general ones of the patient.

The local symptoms are generally easily given in a repertory and so easily found. But the general characteristic symptoms of a remedy are seldom given with any clearness in a repertory; indeed these general characteristic symptoms are made up of several symptoms which must be taken together to give an intelligible idea of the remedy. All of the pains and sensations of Aconite may be found, each under its appropriate heading, in almost any repertory, but where can one find, under one heading, its general characteristics? The characteristics of each remedy are peculiar to it, as are the traits of each individual. When one sees a friend on the street, he recognizes him, even in a crowd, at a glance. He does not need to closely scrutinize each feature, to examine the color of his eyes, the shape of his nose, nor to ascertain his weight or height, etc. All of these points, common to so many persons, are dismissed without a thought, and the friend is recognized by some peculiarity of form or gesture or walk.

So it should be with our remedies; each one should be recognized by its peculiar not by its common symptoms. Aconite, for instance, has many symptoms common to many other remedies; and is therefore only to be prescribed when the patient has the common symptoms *plus* the peculiar one. The great key-note of Aconite is fear; the patient is never cheerful and contented; suppose a patient complains of this symptom, shall Aconite be given? Over one hundred other remedies have it also, in one way or another. Obviously this one symptom, peculiar as it is with this remedy, must be further qualified before it could be safely considered a key-note. So we say the characteristics of Aconite are its anxiety, fear, restlessness, fever, etc. The cough of Aconite and of Squilla are very similar; in one case the patient is restless; in the other, quiet.

In looking up a remedy in a repertory all these points must be considered; but one need look only for the peculiar symptoms of the patient and when a remedy is found which has these, one may safely turn to the *Materia Medica* to make "assurance doubly sure." Let us further illustrate, by supposing a case, of only a few symptoms, for brevity.

The patient is full blooded, well nourished, has dark hair and eyes. Complains of stitches through chest; anxious, labored breathing; painful sensitiveness to contact; sudden sinking of strength; hot, dry skin, fever; burning internally; is irritable, anxious, restless, fearful of death; is worse in evening, lying on left side, etc. These symptoms, collectively, are typical of

Aconite; individually and separately they will be found in a repertory under several drugs. A glance at the *Materia Medica* is needed to give one this complete picture of the remedy.

Many pages might be written upon the use of the repertory; yet it would be merely a repetition of these four points:

1. Examine patient fully, as Hahnemann has directed.

2. Select from the results of this examination the symptoms which are peculiar to the individual under treatment.

3. Seek in a repertory for a remedy (or it may be two or more remedies) having these peculiar symptoms.

4. Consult the *Materia Medica* to be sure the remedy has the peculiar symptoms; thus one prevents errors in repertory from misleading and at same time gets a comprehensive view of the remedy.

As to a comparison of repertories, it seems best for each physician to use one repertory so as to become thoroughly familiar with it in order that he may be able to use it promptly and efficiently. He soon becomes so familiar with its arrangement that he can find anything in it and feels sure no symptom desired can elude his search. When a desired symptom cannot be found in this repertory, then seek for it in every repertory, or thumb the *Materia Medica* until it be found; and when found, make a note of it at once, in the appropriate place in the working repertory, so that forever after that symptom will be readily found. By so entering each new symptom, as it is found, one gets finally a very valuable repertory. A poor repertory, whose arrangement and contents are well known is of more practical value to a prescriber than a more complete repertory which is a *terra incognita.* Using a repertory as a mere index to the *Materia Medica* prevents misprints, errors, and omissions from causing erroneous prescriptions. In many repertories Ars. is printed Arn.; Ang. for Arg., etc., etc.; such errors would readily cause one to use Arnica instead of Arsenic; or Angustura for Argentum, etc., were he to rely solely upon the repertory.

Many physicians compile repertories in MSS., using various arrangements. It would seem more useful to use the working, every-day repertory, as the basis and build upon it, as just suggested.

It will thus be seen that the consideration of the "Use of a Repertory" involves also the consideration of the question, "How to prescribe;" for unless one knows how to prescribe homœopathically he cannot know how to use a repertory. Unless he knows just which symptoms of his patients are to be used to base his prescription upon, he will be at a loss to know what he is to search for in his repertory. If he looks up every symptom, the common as well as the peculiar and characteristic, he will perform much unnecessary labor and most probably become confused and disheartened. By sifting out from the mass of symptoms those which are peculiar and uncommon and seeking for a remedy covering these, one makes his task easier, more certain and insures success; especially so if he refers to his *Materia Medica* to be sure he has the true simillimum.

